# Life Cycle Assessment (LCA) of Particleboard: Investigation of the Environmental Parameters

**DOI:** 10.3390/polym13132043

**Published:** 2021-06-22

**Authors:** Muhammad Aiman Hakim Mohd Azman, Sharizal Ahmad Sobri, Mohd Natashah Norizan, Mohd Nazri Ahmad, Wan Omar Ali Saifuddin Wan Ismail, Kamarul Ariffin Hambali, Mohd Hendra Hairi, Andi Hermawan, Mazlan Mohamed, Pao Ter Teo, Mohammad Radzif Taharin, Noorsidi Aizuddin Mat Noor

**Affiliations:** 1 Advanced Material Research Cluster, Faculty of Bioengineering and Technology, Universiti Malaysia Kelantan, Jeli Campus, Jeli 17600, KTN, Malaysia; aimanah67@gmail.com (M.A.H.M.A.); andi@umk.edu.my (A.H.); mazlan.m@umk.edu.my (M.M.); teopaoter@umk.edu.my (P.T.T.); 2 Geopolymer and Green Technology, Centre of Excellence (CEGeoGTech), Universiti Malaysia Perlis, Kangar 01000, PLS, Malaysia; 3 Faculty of Electronic Engineering Technology, Universiti Malaysia Perlis, Arau 02600, PLS, Malaysia; 4 Environmental Technology Research Centre (ETRC), SIRIM Berhad, No. 1, Persiaran Dato’ Menteri, Seksyen 2, Shah Alam 40700, SGR, Malaysia; mdnazri@sirim.my; 5 Faculty of Innovative Design and Technology, Universiti Sultan Zainal Abidin, Gong Badak Campus, Kuala Nerus 21300, TRG, Malaysia; woasaifuddin@unisza.edu.my; 6 Faculty of Earth Science, Universiti Malaysia Kelantan, Jeli Campus, Jeli 17600, KTN, Malaysia; kamarul@umk.edu.my; 7 Faculty of Electrical Engineering, Universiti Teknikal Malaysia Melaka, Hang Tuah Jaya, Durian Tunggal 76100, MLK, Malaysia; hendra@utem.edu.my; 8 Civil Engineering Programme, Faculty of Engineering, Universiti Malaysia Sabah, Jalan UMS, Kota Kinabalu 88400, SBH, Malaysia; radzif@ums.edu.my; 9 Centre for Real Estate Studies, Department of Real Estate, Faculty of Built Environment and Surveying, Universiti Teknologi Malaysia (UTM), Johor Bahru 81310, JHR, Malaysia; noorsidi@utm.my

**Keywords:** life cycle assessment, environment, wood-based industry, particleboard, Malaysia, openLCA

## Abstract

Particleboard is not entirely a wood replacement but a particular material with its properties, making it more effective at different times than heavy or solid wood. The world’s biggest concern is environmental problems with formaldehyde as a particulate board binder that can lead to human carcinogenic agents. A cradle-to-gate life cycle assessment (LCA) of particleboard production was performed using openLCA software. The impact assessment was carried out according to the software’s features. This preliminary investigation aims to analyze the chemical composition of particleboard and identify its environmental impact. The Fourier-transform infrared spectroscopy (FTIR) system was used to track the functional group of aliphatic hydrocarbons, inorganic phosphates, and main aliphatic alcohols found in particleboards made in Malaysia. Based on the FTIR results, aliphatic groups were found in numerous aggravates that the spectroscopic infrared was likely to experience. The most important vibrational modes were C–H, at approximately 3000 cm^−1^, and –CH deformations around 1460 cm^−1^ and 1380 cm^−1^. Eight effect groups demonstrated that 100% of the input and all analyses produced the same relative outcome. The life cycle of a product is determined by pollution of the air, water, and soil. Thus, particleboard has a minimal impact on the environment, except for global warming.

## 1. Introduction

Globally, particleboard’s growth is on the brink of change, and consumer development is slowly coming to an end worldwide [1]. These developments’ net result is an increase of 1,735,000 m^3^ in particleboard’s potential globally. These rises come from new factories in China, Malaysia, and Thailand—the driving force behind recent particleboard production. Two factors are expected to significantly impact the furniture industry: changing the way of life of the older generation and increasing the young demographic. The demand for lightweight and ready-made furniture is growing. Therefore, the particleboard market is essential, as ready-made furniture is generally manufactured using particleboard because it is less expensive than wooden boards or fiberboards. As a result of these factors, entrepreneurs must prepare for significant future changes. The market for particleboards depends on a significant increase in the development of the reception and use of particleboards in furniture and construction activities.

Pressure on businesses has risen due to the decline in or elimination of the ecological impact of products. It is important to follow a life cycle strategy to enhance the ecological presentation of objects [2]. Non-exclusive life cycle assessment (LCA) knowledge of particleboards is also important. For example, in the LCA of a wooden cabinet, the particleboard age cycle began to have the most significant natural impact [3]. Life cycle methods assessment (LCMA) has been developed to classify the most critical adverse consequences and key hotspots such as processes and methods that lead directly to environmental impacts.

A few LCA contextual analyses of particleboard formation have been produced and published, for example, on particleboards in Spain [4], the USA [5,6], Brazil [7], and Iran [8]. Nevertheless, no LCA inquiries or analyses of Malaysian particleboard have been released. Wilson [5] led a far-reaching analysis on the life cycle inventory (LCI) of particleboard manufacturing in the US by examining the various processes in the particleboard production chain, from raw materials procurement to manufacturing the finished components, oils, power consumption, and pressing of the commodity. A further study of particleboard formation LCA was led by Pueetman et al. [6] in the USA in 2013. The findings revealed that backwoods properties contributed fewer outflows than assembly processes such as the furnace, drying, and particleboard pressing methods, functioning, and power as non-renewable energy sources utilized and resin form. In addition to that, Silva et al. [7] guided a point-by-point review on LCA of medium-density particleboard in Brazil, considering backwoods and urban architecture processes separately or jointly. Kouchaki-Penchah et al. [8] released a continuing, far-reaching report on LCA particleboard manufacturing in Iran.

The key purpose of this research is to perform a particleboard life cycle assessment (LCA) in Malaysia. This preliminary research is related to the environmental impacts of particleboard, which are linked to the following objectives: (a) to analyze the particleboard’s chemical quality; and (b) to identify the environmental impact of the particleboard. This research further reflects on examining the practical effect of particleboard output over the whole life cycle. The data must be established and obtained from the particleboard analysis phase using the LCA system. The environmental and human effects of particleboard manufacturing must also be understood and remembered. This study showed that LCA is essential and of excellent assistance to a developing country such as Malaysia. Essentially, LCA would help educate a person about the importance of a “natural” or eco-friendly environment in the future or the next decade. Furthermore, LCA often requires each organization to identify the environmental impact of similar or unique items.

Life cycle assessment (LCA) is a precious method for recognizing ecological viewpoints and possible effects relevant to goods, systems, or administration [9]. The technique consists of a stock of fair vitality and material sources discharged to an area. In addition, it describes the possible environmental effects correlated with agricultural inputs. Next, a study of the outcome of the decision on the right choice of human well-being and the ecological effects of goods is carried out. LCA has been used to research various products and processes, from aircraft motors, bottles, food tables, tablets, garbage reduction, and remediation strategies [10].

For some common products, life cycle assessment considers that the production of basic substances is expected to produce a drug, intermediates, and, ultimately, the product itself. It also includes packaging, the transport of raw materials, intermediates, and the element that contains the component and the storage of the item after use. This situation is referred to as the “cradle-to-grave” appraisal [11].

The ISO 14040/14044 standard defines LCA as an aggregation and assessment of knowledge inputs, yields, and potential natural effects of an entity structure during its life cycle (ISO, 2006). In this description, there is a substantial effect on estimating the fundamental influence in LCA. Evaluation of the life cycle effect is described as being followed by an understanding and evaluation of the importance and relevance of the possible natural consequences of the commodity mechanism. ISO 14040/14044 accepts the necessary classification and recognition elements.

Particleboard is a porous board composite composed of cellulose particles of different sizes bound together with a synthetic resin or a heat and pressure binder [8]. Particle structure, resin quantities, board thickness, density, and production methods can be adjusted to manufacture goods appropriate for a specific end use [5,6]. Urea-formaldehyde is usually found in the standard particleboard as a solvent. The amount of adhesive in particleboard is 10%, and much of the painted board items are included in the group of surface material contamination. The description of the substance includes the criteria for emissions of the construction materials planned for the interior and their quality.

Formaldehyde, however, has been identified as carcinogenic. Risk assessment of the potential health and environmental impacts that the production, use, and disposal of particleboards may give rise to provides the required information on both the potential for exposure to materials and the potential for uncovered impacts, such as toxicity or greenhouse gas (GHG) emissions. In this research attempt, the particleboard topic provides exposure in experiments designed to determine the ecological consequences of particleboard processing by utilizing the LCA methodology.

Additionally, openLCA is one of the open-source LCA and sustainability evaluation tools developed by GreenDelta [12]. As an open-source application, it is widely accessible with no license price [13]. OpenLCA allows for faster recording of tests with the most extensive information structure accessible to LCA. Numerous company clients rely on the proven and internationally utilized LCA database (i.e., GABI or Ecoinvent), which can be used in openLCA. Gabi or Ecoinvent is a database that is unreservedly used in openLCA programming [14]. OpenLCA is one of the tools that was used to classify environmental effects in this research. The use of the software included a thorough understanding of the relationship between each unit of the operation. The primary problems emerging from the collection of knowledge are the obstacles to repositories and the absence of clarity on their material [15].

## 2. Materials and Methods

The materials (i.e., particleboards) used for this analysis were obtained from manufacturing areas in Kota Bharu in the state of Kelantan, Malaysia. The particleboard’s dimensions are 30 cm broad, 12 cm wide, and 1.5 cm thick. The color of the laminate particleboard is brown-choc, brown-black, brown-white, white, and beige. Chemical compounds which have been used are formaldehyde-based and are intended for adhesive use. As these adhesives have been synthesized for particleboards, the study of such environmental effects should be included in the LCA research [7]. Volume was considered as the functional unit for the outputs in this investigation. As a result, the functional unit for environmental parameters was standardized in this study. The functional unit chosen for this investigation was 1.0 m^3^ of the finished particleboard to facilitate comparability with other works.

This work was performed in compliance with the ISO 14044 standard. There are four stages of a life cycle evaluation analysis. As mentioned above, the key raw material in this work is particleboard manufactured from wood chips or sawdust and some solvent, such as melamine urea-formaldehyde. Phases of LCA are as follows:

- Goal and scope definition phase;
- Inventory analysis phase;
- Impact assessment phase;
- Interpretation phase.

The Life Cycle Assessment software program is available to LCA and was used to conduct the impact assessment process.

The aim and context of the research are as follows: the goal of the analysis was to examine the particleboard chemical material’s environmental impact.

**Figure 1:**
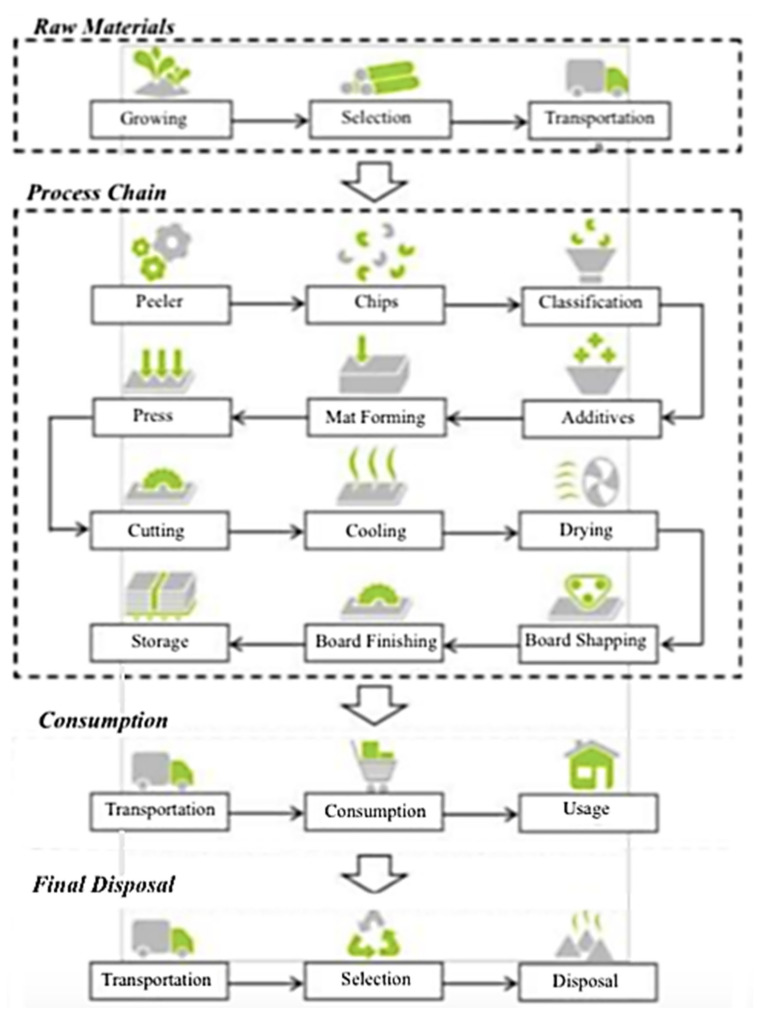
System boundary of particleboard life cycle from cradle to gate.

System boundaries: This study focused only on environmental impacts due to the limitation of time and data details. The cradle-to-gate technique was used to define the boundaries of a product system. Cradle-to-gate is a simplified assessment of a product’s life cycle that focuses exclusively on the period from resource extraction (cradle) to factory gate (i.e., before it is transported to the consumer). Due to the difficulty of collecting data on the distribution, usage, and disposal of roofing tiles, this study omitted these topics. The system boundary for the particleboard life cycle is depicted in Figure 1. Particleboard is made by combining wood particles or flakes with a resin and molding the mixture into a sheet. The raw materials are fed into a disc chipper with four to sixteen radially aligned blades. Disk chippers produce chips that are more uniform in shape and size than other types of wood chippers. After drying, the particles are filtered to remove any large or undersized particles. The particles are then sprayed with resin in the form of a fine mist. The technique employs a variety of resins. In terms of cost and convenience of usage, amino-formaldehyde-based resins perform the best. Water resistance is provided by urea melamine resins, with more melamine providing greater resistance. They are primarily used outside, with the colored resin darkening the panel. Resorcinol resins can be combined with phenolic resins to improve panel characteristics further; however, this technique is more commonly utilized in marine plywood applications. Other chemicals such as wax, dyes, wetting agents, and release agents are used in panel production to aid processing or to make the end product resistant to water, fire, or insects. After passing through a mist of resin sufficient to coat all surfaces, the particles are stacked into a continuous carpet. This “carpet” is then cut into discrete, rectangular “blankets,” which are compressed in a cold press. The flakes are weighed on a scale and spread by revolving rakes. The flakes in graded-density particleboard are dispersed by an air jet that propels finer particles further than coarse particles. Two of these jets, when reversed, allow particles to accumulate from fine to coarse and back to fine. Cold compression is used to lower the thickness of the created sheets and make them easier to carry. Later, they are crushed again at pressures ranging from 2 to 3 megapascals (290 to 440 psi) and temperatures ranging from 140 to 220 °C (284 to 428 °F) to set and cure the adhesive. The entire process is monitored to ensure that the board is the correct size, density, and uniformity. After that, the boards are cooled, cut, and sanded. They can then be offered as raw board or with a surface improvement such as a wood veneer or laminate surface.

Inventory analysis phase: This research focused only on particleboard. Inventory data for the chemical content were collected during the particleboard analysis process. In this step, Fourier-transform infrared spectroscopy (FTIR) was used to classify the particleboard compound. The particleboard was first cut into smaller pieces. The particleboard’s smaller parts were processed into powder form using a wood grinder before conducting core studies. Eventually, the powder was examined using FTIR to achieve a functional group and a compound found in the particleboard.

Impact assessment phase: The openLCA program was used to analyze the chemical content’s impacts on the particleboard. The entire database was passed to the program, and the final result for the LCA of the particleboards was returned.

Interpretation phase: In this step, the analysis was interpreted and linked to the purpose and scope of the study; therefore, the interpretation was drawn, the restriction was established, and the advice was based on the findings of the phases of the LCA. This process often defined dangerous or particleboard products. According to Woolridge et al. [16], most of these environmental parameters are listed for assessment:

- Global warming (GWP 100a);
- Depletion of abiotic resources;
- Acidification;
- Eutrophication;
- Freshwater aquatic ecotoxicity;
- Human toxicity;
- Marine aquatic ecotoxicity;
- Ozone layer depletion;
- Photochemical oxidation;
- Terrestrial ecotoxicity.

Fourier-transform infrared spectroscopy (FTIR) is an analytical technique used to identify organic and, in some instances, inorganic materials. This method tests the absorption of infrared radiation from the spectrum by the material of the specimen. Infrared absorption bands are used for the characterization of molecular materials and structures. When a material is irradiated with infrared (IR) radiation, the IR radiation obtained usually excites molecules to a higher vibrational point. The energy difference between the at-rest and excited vibrational conditions determines the wavelength of light emitted by a single molecule. Its wavelengths represent the material’s molecular structure. The FTIR characterization was carried out using a small amount of particleboard powder. The determination of the functional group and spectral properties of the individual nanoparticles was conducted using the FTIR model Thermo Scientific TM iD7 (Fisher Scientific (M) Sdn. Bhd., Shah Alam, Malaysia), with a single-bounce attenuated total reflectance (ATR), and a scan range between 400 and 4000 cm^−1^, at the Faculty of Bioengineering and Technology, Universiti Malaysia Kelantan, Jeli Campus in Kelantan, Malaysia.

## 3. Results and Discussion

**Figure 2:**
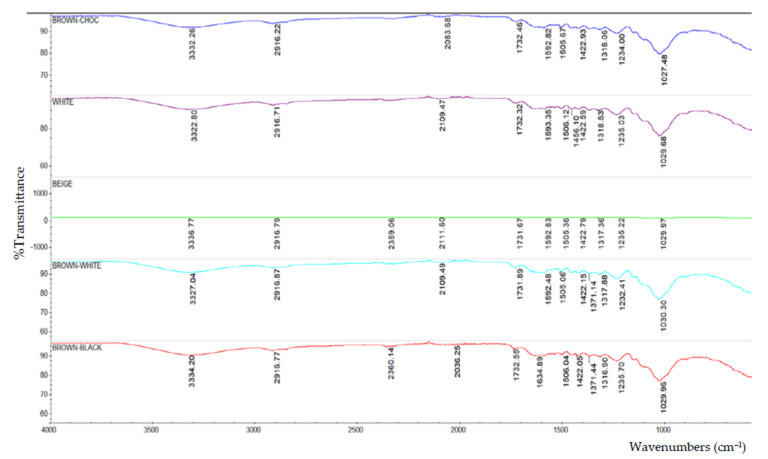
The peak in the FTIR analysis for different types of particleboard.

**Figure 3:**
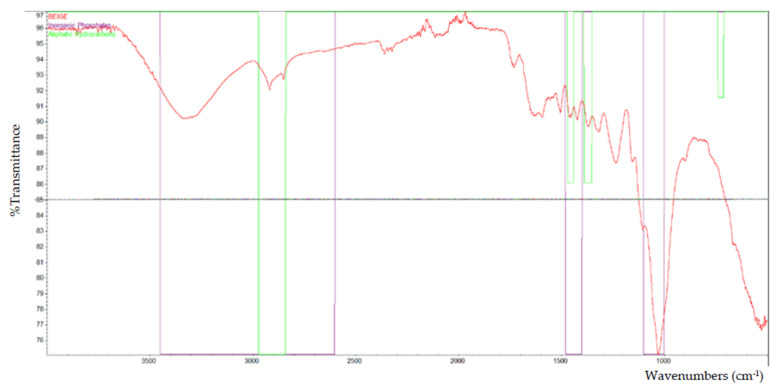
The functional group that is present in laminated (beige) particleboard.

**Figure 4:**
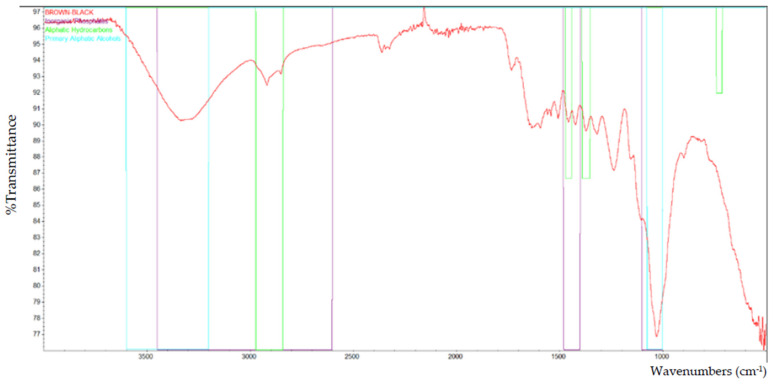
The functional group that is present in laminated (brown-black) particleboard.

**Figure 5:**
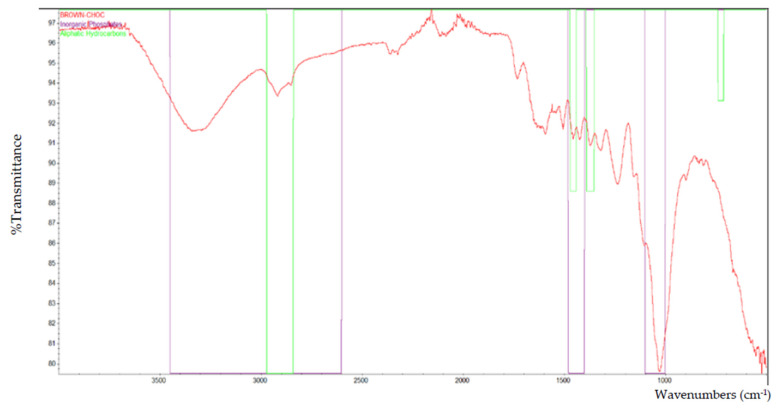
The functional group that is present in laminated (brown-choc) particleboard.

**Figure 6:**
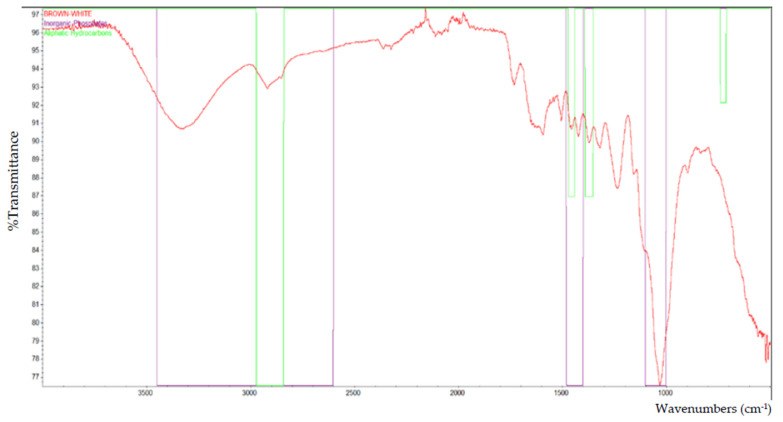
The functional group that is present in laminated (brown-white) particleboard.

**Figure 7:**
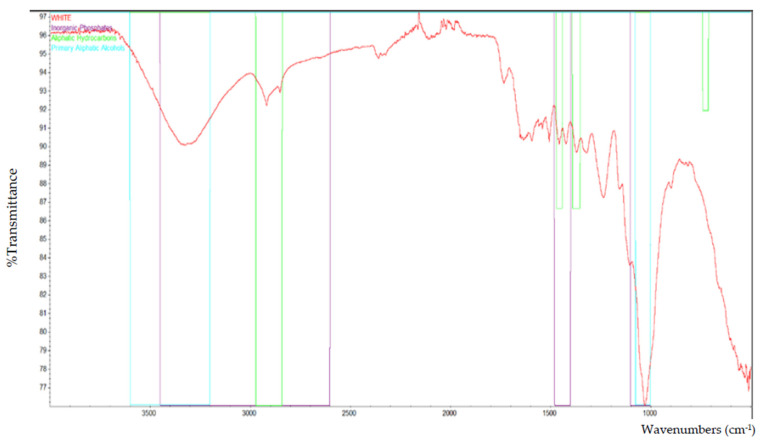
The functional group that is present in laminated (white) particleboard.

Based on the FTIR analysis, Figure 2 indicates the peak for all of the particleboard samples. Inorganic phosphate, aliphatic hydrocarbons, and primary aliphatic alcohol were among the materials discovered in particleboard. Figure 3, Figure 4, Figure 5, Figure 6 and Figure 7 are mostly composed of inorganic phosphate and aliphatic hydrocarbons, with the exception of Figure 4 and Figure 7, which have both features listed as previously mentioned.

Inorganic phosphates had significant trademark spectra. There were two groups of about 1000 cm^−1^ and 550 cm^−1^. Water groups are also commonly found around 3400 cm^−1^ and 1640 cm^−1^. All figures (i.e., Figure 3, Figure 4, Figure 5, Figure 6 and Figure 7) indicate that the band peaks at 3336.77 cm^−1^, 3334.20 cm^−1^, 3332.26 cm^−1^, 3327.04 cm^−1^, and 3322.80 cm^−1^. The length of the stretch is high.

In other words, aliphatic groups were found in numerous aggravates that the spectroscopic infrared was likely to experience. The most important vibrational modes were C–H, at approximately 3000 cm^−1^, and –CH deformations around 1460 cm^−1^ and 1380 cm^−1^. The peaks selected based on these figures (i.e., Figure 3, Figure 4, Figure 5, Figure 6 and Figure 7) are 2916.79 cm^−1^, 2916.77 cm^−1^, 2916.22 cm^−1^, 2916.87 cm^−1^, and 2916.71 cm^−1^, which were the peaks shown by the aliphatic hydrocarbon functional groups—the atom directly connected to the aliphatic groups that cause critical movements from the standard frequencies. Specifically, neighboring molecules with intense electro-pessimism can shift the band areas to higher frequencies.

Furthermore, alcohols contain hydroxyl (–OH) mixtures for primary aliphatic alcohols. These mixtures were delegated as essential, auxiliary, or tertiary, as indicated by the quantity of other carbon attached to the oxygen-bound carbon.

Alcohols produce an unusual polar –OH range. This caused the retention of hydrogen between atoms at the condensed level. Due to this hydrogen retention, alcohol breakpoints were much larger than alkane compared to a comparable number of carbon molecules.

(1) –C–O stretching and –OH deformation vibrations: 

Primary alcohols: 1050 cm^−1^;

Secondary alcohols: 1100 cm^−1^;

Tertiary alcohols: 1150 cm^−1^;

Phenols: 1200 cm^−1^.

(2) –OH stretching frequencies (free –OH form): 

Primary alcohols: 3643–3630 cm^−1^;

Secondary alcohols: 3635–3620 cm^−1^;

Tertiary alcohols: 3620–3600 cm^−1^;

Phenols: 3612–3593 cm^−1^.

As stated above, –C–O stretching and –OH deformation vibrations of primary alcohols are found at 1050 cm^−1^. The amount similar to this is 1029.68 cm^−1^ in Figure 4 and 1029.96 cm^−1^ in Figure 7, as the FTIR test results show only those values containing primary aliphatic alcohols. Since –OH extending frequencies and the free –OH shape cannot be defined due to the FTIR analysis findings, only 3500.42 cm^−1^ and 3520.46 cm^−1^ for both figures represent the maximum peaks observed.

Table 1, Table 2, Table 3, Table 4 and Table 5 show the substances that were detected or identified in particleboard. Any of these materials were passed to the openLCA program for further review. The primary additive used in particleboard production was formaldehyde, and several of the compounds typically used to produce laminated particleboard. Laminated particleboard for brown-choc in Table 1, white in Table 2, and brown-black in Table 5 shows the same compound but at a significantly different percentage of the compounds.

Table 6, Table 7 and Table 8 provide a summary of all inputs and outputs for on-site particleboard growth. Such inputs were provided by 1.0 m^3^ of particleboard.

**Figure 8:**
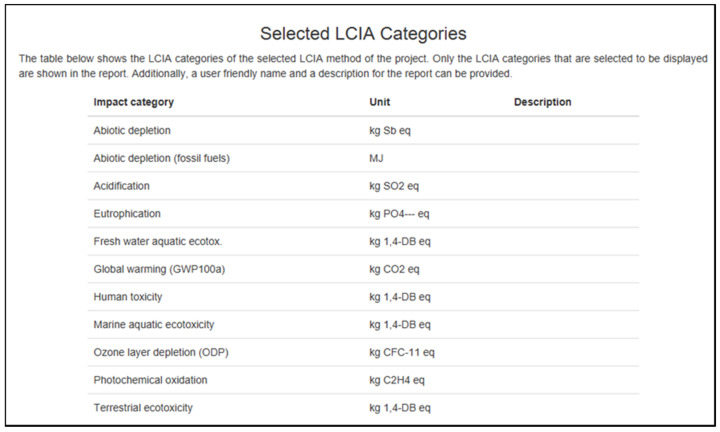
The life cycle inventory analysis (LCIA) categories of the project.

The input and output for both tables were generated and referred to several of the studies by Wilson [5] and Puettman et al. [6]. Nonetheless, owing to the report restriction, specific details cannot be used, and complete detail on particleboards’ development is confidential. The risk groups (see Figure 8) identified are climate change, abiotic degradation, acidification, eutrophication, freshwater aquatic ecotoxicity, human toxicity, marine aquatic ecotoxicity, ozone depletion, photochemical oxidation, and terrestrial ecotoxicity.

**Figure 9:**
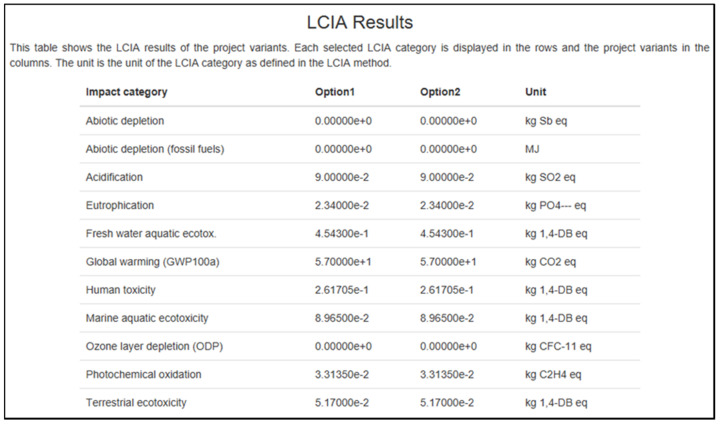
The results from openLCA software for brown-choc, white, and brown-black types.

Based on Figure 9, this table displays the results that were obtained from the openLCA program. Option 1 and option 2 are implied in the chart. For choice 1, the projected results were with the usage of 1.0 m^3^ of particleboard. Subsequently, further study using alternative 2, which is the calculation, was increased to 10 m^3^. This figure indicates that despite many particleboard changes, the figure always returns the same outcome as option 1. Zero results mean that the particleboard will not damage the environment, such as the loss of abiotic material and depletion of the ozone layer.

**Figure 10:**
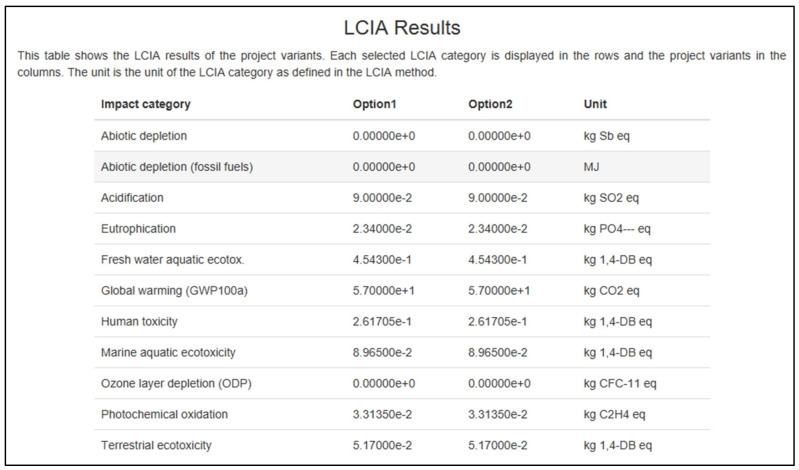
The results from openLCA software for beige type.

**Figure 11:**
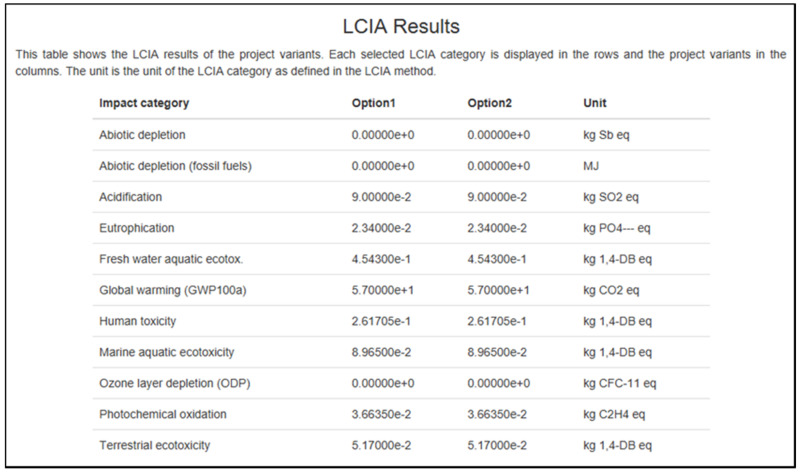
The results from openLCA software for brown-white type.

However, other types of effect have been found to have an economic influence, particularly global warming (i.e., the maximum at 57 kg of CO_2_ eq., and this is just for one day). As many sessions are conducted in a day, month, and year, it would significantly affect our climate. Environmental warming is caused by greenhouse gas (GHG) pollution from the economy. The phytochemical oxidation component results are shown in Figure 9 and Figure 10. Figure 11 shows slightly different, which is 0.037 kg C_2_H_4_ eq. added to the other 0.033 kg of C_2_H_4_ eq. from the results in Figure 9 and Figure 10.

Based on the openLCA results, the inputs that can be introduced into the flow component are urea-formaldehyde. Simultaneously, the output that can be measured is the volume of formaldehyde that can be emitted into the air from the processing of the particleboard. Data inputs and outputs can be formulated on a computer, depending on the availability of openLCA features. Product life cycle results rely on pollution of air, water, and soil. Based on the result, except for global warming, particleboard is only marginally detrimental to the environment.

**Figure 12:**
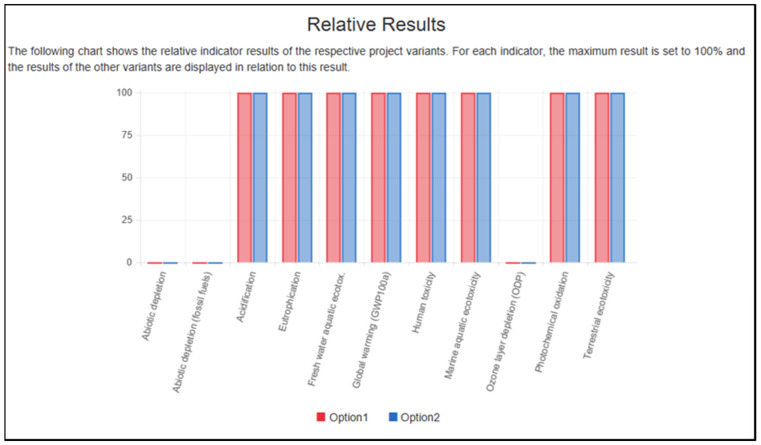
The graph generated from openLCA software.

Based on Figure 12, the tests are for the minimal level of particleboard. The x-axis shows the eleven impact categories, while the y-axis scale adjusts depending on the particleboard dimension. The openLCA program conducted the calculation and converted all the figures into a different percentage emission function. Eight effect groups revealed that 100% of the input and all the analyses returned the same relative outcome. This ensures that input and output elements, such as chemical emissions, transport, wood fuel water, energy, formaldehyde (adhesive), and wood residues, have entirely contributed to a cradle-to-gate environmental impact assessment.

Based on these results, any company will create a monopoly on the manufacture of particleboards by processing that has had a significant environmental impact, and this is also aligned with the findings of Therasme et al. [17]. Of note, the carbon footprint was lowered by calculating the amount of greenhouse gas (GHG) emissions that included carbon dioxide, carbon monoxide, methane, and others that may mitigate the impact of global warming [18,19].

Information collection may also be conducted through a detailed survey questionnaire by the organization to report all inputs of goods, fuels, and energy and all outputs of resources, co-products, and pollutants of land, water, and wastewater. Test knowledge must be tested for accuracy by finding outliers and conducting mass and energy balances. Each mill’s details must be translated to a manufacturing base unit and, in this case, to a cubic meter (1.0 m^3^). This method of data collection may be used later for further research purposes.

## 4. Conclusions

Investigations were carried out with life cycle assessment (LCA) application to particleboards from the Malaysian wood-based industry. With this specific particleboard made from wood chips or sawdust and a popular additive, urea-formaldehyde, this engineered wood material is quite significant given that it marginally emits hazardous effects. This study’s objectives were all met. The first was to analyze the chemical composition of the particleboards. This goal was accomplished using FTIR for the functional category of aliphatic hydrocarbons, inorganic phosphates, and main aliphatic alcohols in the particleboards. The second goal was to identify the environmental impacts of the particleboard. This was achieved using the openLCA application, which shows the preliminary findings of this research and calculates the input and performance that contribute to the cradle-to-gate environmental impact evaluation.

The LCA methodology assists in pointing out the practical approach by providing clearer information to LCA practitioners, technologists, engineers, and stakeholders. It provides a process of multi-partner dialogue and transparent collaboration with companies and governments, from the community’s impetus to the state and general frameworks of economic advancement. This inquiry creates a far-reaching framework for the particleboard LCI. The information should be used to justify any LCA of its environmental display to enhance handling or comparison with different materials. Similar framework conditions and energy values must be used when contrasting or comparing the data in this investigation with other processes and products.

Due to the lack of external funding, this research focuses only on the environmental implications identified during life cycle assessment. However, this research may provide valuable inputs for future life cycle assessment projects. In future work, social, economic, financial, and environmental elements of life cycle evaluation should be included in the research comprehensively. Additional investigations are recommended in order to gain access to the LCI particleboard database; particularly noteworthy is a further examination of FTIR findings in LCIA. Aliphatic groups were found in numerous aggravates that the spectroscopic infrared was likely to experience. The most important vibrational modes were C–H, at approximately 3000 cm^−1^, and –CH deformations around 1460 cm^−1^ and 1380 cm^−1^. Based on the preliminary LCIA, eight effect groups demonstrated that 100% of the input and all analyses produced the same relative result. This assures that all input and output factors, such as chemical emissions, transportation, wood fuel water, energy, formaldehyde (adhesive), and wood residues, have contributed fully to a cradle-to-gate environmental impact assessment. Extensive research on the impact of replacing wood with non-renewable energy sources and efforts to conduct a carbon or particleboard flux investigation beyond the product gate should be explored. This covers usage, disposal, and reuse.

Further research to investigate the electricity consumption within the production of particleboard should be undertaken. Teow et al. [20] compared the environmental consequences of four combinations of integrated membrane filtration systems to treat aerobically digested palm oil mill effluent using the LCA methodology. According to previous studies, electricity consumption is the primary cause contributing to the majority of such categories (50–77%) as carbon dioxide (CO_2_), sulfur dioxide (SO_2_), nitrogen oxides, and volatile mercury emissions during the combustion of fossil fuels. By this study, energy usage contributes to environmental impacts across all life cycle impact categories. This study is a good reference for investigating electricity consumption.

Another interesting work of Richard et al. [21] investigated the economic cost and environmental burdens in municipal solid wastes (MSW) management. Compared to the other scenarios, one of the solutions, which is a combination of recycling, composting, and landfilling, had the lowest economic cost and environmental impact in most categories. The sensitivity study results showed that boosting diesel usage, lowering methane emissions into the atmosphere, and raising paper and plastic recycling rates would lessen the total environmental consequences across all scenarios. This is also a useful reference when evaluating various particleboard manufacturing processes from an economic standpoint.

## Figures and Tables

**Table 1:** The results of FTIR analysis for laminated (brown-choc) particleboard.

Compound	% Matched
Cellophane	63.98
Cellulose	59.62
Dextrose monohydrate powder	51.52
Cellophane	50.15
Isomaltose approximtaely 99%	48.18
Chondroitin sulfate grade III SOD	46.94
OPIUM powder in KBR	46.76
Formaldehyde aqueous solution	45.28
Dextrose anhydrous powder in KBR	44.28
6-Deoxy-D-Glucose crystalline	40.42

**Table 2:** The results of FTIR analysis for laminated (white) particleboard.

Compound	% Matched
Cellophane	64.54
Cellulose	56.55
Dextrose monohydrate powder	51.52
Cellophane	50.83
Isomaltose approximately 99%	48.03
Formaldehyde aqueous solution	47.71
OPIUM powder in KBR	47.31
Chondroitin sulfate grade III SOD	47.00
Dextrose anhydrous powder in KBR	44.92
6-Deoxy-D-Glucose crystalline	39.60

**Table 3:** The results of FTIR analysis for laminated (beige) particleboard.

Compound	% Matched
Cellophane	65.16
Cellulose	57.16
Dextrose monohydrate powder	54.06
Cellophane	52.70
Isomaltose approximately 99%	49.36
Formaldehyde aqueous solution	48.77
OPIUM powder in KBR	47.95
Dextrose anhydrous powder in KBR	46.33
Chondroitin sulfate grade III SOD	45.73
Benzyl alcohol, 99%	40.57

**Table 4:** The results of FTIR analysis for laminated (brown-white) particleboard.

Compound	% Matched
Cellophane	65.11
Cellulose	55.88
Dextrose monohydrate powder	50.01
Cellophan	48.96
Chondroitin sulfate grade III SOD	46.49
OPIUM powder in KBR	46.39
Isomaltose approximately 99%	45.59
Formaldehyde aqueous solution	44.96
Dextrose anhydrous powder in KBR	41.40
Methanol	38.23

**Table 5:** The results of FTIR analysis for laminated (brown-black) particleboard.

Compound	% Matched
Cellophane	62.69
Cellulose	55.25
Dextrose monohydrate powder	51.84
Cellophane	48.76
Chondroitin sulfate grade III SOD	47.95
OPIUM powder in KBR	47.73
Isomaltose approximately 99%	47.03
Formaldehyde aqueous solution	45.73
Dextrose anhydrous powder in KBR	42.73
6-Deoxy-D-Glucose crystalline	39.20

**Table 6:** Inputs and outputs for the processing of 1.0 m^3^ particleboard for brown-white type.

Production Data	Unit	Unit/m^3^
Inputs	-	-
Materials	-	-
Wood residue	kg	672
Urea-formaldehyde	kg	68
Slack wax	kg	2.5
Sodium sulfate	kg	7.2
Polyethene	kg	0.46
Electricity	-	-
Electricity	MJ	569
Fuels	-	-
Natural gas	M^3^	30
Diesel	L	0.26
Water use	-	-
Municipal water source	L	304
Outputs	-	-
Particleboards	kg/m^3^	746
Co-product	-	-
Wood fuel (sold)	kg/m^3^	5.2
Emission to air	-	-
Carbon dioxide, biogenic	kg	56
Carbon dioxide, fossil	kg	57
Carbon monoxide	kg	0.17
Nitrogen oxides	kg	0.18
Sulfur oxide	kg	0.006
Formaldehyde	kg	0.055
Methanol	kg	0.025
Emission to water	-	-
Suspended solids	kg	0.01
Emission to land	-	-
Wood waste	kg	0.4
Wood ash, at boiler	kg	0.1

**Table 7:** Inputs and outputs for the processing of 1.0 m^3^ particleboard for beige type.

Production Data	Unit	Unit/m^3^
Inputs	-	-
Materials	-	-
Wood residue	kg	672
Urea-formaldehyde	kg	68
Slack wax	kg	2.5
Sodium sulfate	kg	7.2
Polyethene	kg	0.46
Electricity	-	-
Electricity	MJ	569
Fuels	-	-
Natural gas	M^3^	30
Diesel	L	0.26
Water use	-	-
Municipal water source	L	304
Outputs	-	-
Particleboards	kg/m^3^	746
Co-product	-	-
Wood fuel (sold)	kg/m^3^	5.2
Emission to air	-	-
Carbon dioxide, biogenic	kg	56
Carbon dioxide, fossil	kg	57
Carbon monoxide	kg	0.17
Nitrogen oxides	kg	0.18
Sulfur oxide	kg	0.006
Formaldehyde	kg	0.055
Benzyl alcohol	kg	0.040
Emission to water	-	-
Suspended solids	kg	0.01
Emission to land	-	-
Wood waste	kg	0.4
Wood ash, at boiler	kg	0.1

**Table 8:** Inputs and outputs for the processing of 1.0 m^3^ particleboard for brown-black, white, and brown-choc types.

Production Data	Unit	Unit/m^3^
Inputs	-	-
Materials	-	-
Wood residue	kg	672
Urea-formaldehyde	kg	68
Slack wax	kg	2.5
Sodium sulfate	kg	7.2
Polyethene	kg	0.46
Electricity	-	-
Electricity	MJ	569
Fuels	-	-
Natural gas	M^3^	30
Diesel	L	0.26
Water use	-	-
Municipal water source	L	304
Outputs	-	-
Particleboards	kg/m^3^	746
Co-product	-	-
Wood fuel (sold)	kg/m^3^	5.2
Emission to air	-	-
Carbon dioxide, biogenic	kg	56
Carbon dioxide, fossil	kg	57
Carbon monoxide	kg	0.17
Nitrogen oxides	kg	0.18
Sulfur oxide	kg	0.006
Formaldehyde	kg	0.055
Emission to water	-	-
Suspended solids	kg	0.01
Emission to land	-	-
Wood waste	kg	0.4
Wood ash, at boiler	kg	0.1

## Data Availability

The data presented in this study are available on request from the corresponding authors.
